# Radiographic and clinical outcomes of surgical treatment of Kümmell’s disease with thoracolumbar kyphosis: a minimal two-year follow-up

**DOI:** 10.1186/s12891-021-04640-8

**Published:** 2021-09-06

**Authors:** Hao Cheng, Guo-dong Wang, Tao Li, Xiao-yang Liu, Jian-min Sun

**Affiliations:** 1 Department of Spine Surgery, Shandong Provincial Hospital, Cheeloo College of Medicine, Shandong University, Jinan, Shandong China; 2 Department of Spine Surgery, Jinhua Municipal Central Hospital, Jinhua, Zhejiang China; 3 Department of Spine Surgery, Shandong Provincial Hospital Affiliated to Shandong First Medical University, Jinan, Shandong China

**Keywords:** Osteoporotic vertebral compression fracture, Kümmell’s disease, Kyphosis, Imbalance, Spine, Pain,treatment

## Abstract

**Background:**

Up to now in the surgical treatment of Kümmell’s disease combined with thoracolumbar kyphosis, little research has focused on the evaluation of the imaging and clinical outcomes of restoring the normal alignment and sagittal balance of the spine. This study aimed to evaluate the short to mid-term radiographic and clinical outcomes in the treatment of Kümmell’s disease with thoracolumbar kyphosis.

**Methods:**

From February 2016 to May 2018, 30 cases of Kümmell’s disease with thoracolumbar kyphosis were divided into group A and B according to whether the kyphosis was combined with neurological deficits. All of the cases underwent surgical treatment to regain the normal spinal alignment and sagittal balance. The radiographic outcomes and clinical outcomes of the cases were retrospectively evaluated. The sagittal imaging parameters including sagittal vertebral axis (SVA),thoracic kyphosis (TK),thoracolumbar kyphosis (TLK),lumbar lordosis (LL),pelvic incidence (PI),pelvic tilt (PT),and sacral slope (SS) before operation,immediately after operation,and the last follow-up of each case were measured and evaluated. The clinical results included the Oswestry Disability Index (ODI) and the Numerical Rating Scale (NRS) of the two groups. Statistical software SPSS21.0 was used to analyze the data.

**Results:**

In group A: Mean SVA before operation was 75 mm and 26.7 mm at the final postoperative evaluation (*P* = 0.000); Mean TLK before operation was 39°, and 7.1° at the final postoperative evaluation (*P* = 0.000); Mean NRS before operation was 4.7, compared with 0.9 at the final postoperative evaluation (*P* = 0.000). In group B: Mean preoperative SVA was 62.5 mm and decreases to 30.7 mm at the final postoperative evaluation (*P* = 0.000); Mean TLK before operation was 33°, and 9.7° 2 years post-operation (*P* = 0.000); Mean NRS prior to surgery was 4.0, and 0.8 at the last follow-up evaluation (*P* = 0.000). The improvement of the NRS scores of groups A and B was related to the improvement of the cobb angle (*P* = 0.020); (*P* = 0.009) respectively.

**Conclusion:**

In the treatment of Kümmell’s disease with thoracolumbar kyphosis,to restore the normal alignment and sagittal balance can obtain a satisfactory radiographic and clinical short and medium-term effects.

## Background

Since Kümmell’s disease was first proposed by Hermann Kümmell in 1895,it has been gradually recognized and understood by spinal surgeons [1]. With the aging of the population, Kümmell’s disease,as a complication of osteoporotic vertebral compression fracture (OVCF) [2–5], also shows increased incidence [6, 7]. At present, Kümmell’s disease is defined as ischemic necrosis of the vertebral body after minor spinal injury [5, 6, 8]. As the disease progresses, the injured vertebra gradually collapse, and tend to become wedge shaped due to bone necrosis, followed by kyphosis deformity of the spine.

The treatment of Kümmell’s disease is still challenging for most spinal surgeons. One reason is that there are several different stages of progression of Kümmell’s disease, but the choice of surgical methods for cases of different stages is still controversial [3–5, 9–11]. Especially for the patients with kyphosis,most opinions agree that in the treatment of kyphosis with neurological deficits the injured vertebrae should be removed by posterior vertebral column resection (VCR) to relieve spinal cord compression, then kyphosis should be corrected and spinal stability should be reconstructed [12–17], so as to obtain better clinical effects. However, as for kyphosis with no neurological impairment, most of the literature focus on eliminating the unstable factors of injured vertebra, so as to relieve local chronic and refractory lower back pain [4, 18–21].

Previous theories of Kümmell’s disease suggest that there are two different types of pain, acute pain secondary to a fresh fracture at an early stage, and a chronic low back pain that develops gradually after a plateau of weeks or months [1–3, 6–8]. As for the causes of chronic pain in the later stage, most of the literature indicates that it is due to bone necrosis, fracture nonunion, and instability caused by local micro-motion of the injured vertebra [6, 7, 9, 11, 14]. As the understanding of the spine sagittal balance has deepened, it has gradually been realized that a good spine sagittal balance could help the lower back muscles do a minimal work to maintain a balanced posture [22, 23]. Conversely, the advancement of the gravity line caused by long-term kyphotic deformity of the spine will not only change the stress of the corresponding segment on the intervertebral disc, facet joints, and other tissue structures, but also will further accelerate the degeneration of the spine leading to the appearance of discogenic low back pain. At the same time, long-term kyphosis will cause strain and degeneration of the back extensors, which will further aggravate the sagittal imbalance [24, 25]. It has been reported that patients with chronic kyphotic deformities or sagittal imbalance have a higher proportion of long-term refractory pain in the back [26, 27].

Kümmell’s disease occurs frequently in thoracic and lumbar segments [28]. And kyphosis secondary in the above segments often leads to the sagittal imbalance, which can easily lead to further aggravation of existing low back pain. Although more and more spine surgeons have begun to pay attention to sagittal spinal balance in the past 10 years, there are few studies published in the literature about surgical treatment of thoracolumbar kyphosis.

A fact that almost all spine surgeons accept is that the normal physiological angle of the thoracolumbar segment is 0° [29, 30]. And it has also been reported in the literature that patients with thoracolumbar sagittal imbalance usually experienced a significant improvement in low back pain after corrective surgery [26, 31]. Considering the factors above for the further evaluation of postoperative imaging and clinical effects of Kümmell’s disease with thoracolumbar kyphosis, Shandong Provincial Hospital conducted this retrospective study.

## Methods

### Ethics

This retrospective study was approved by the Medical Ethics Committee of Shandong Provincial Hospital and was conducted in accordance with the guidelines of the Declaration of Helsinki. Written informed consent was obtained from each patient.

### Selection of patients and clinical and imaging evaluation criteria

From February 2016 to May 2018, 30 cases of Kümmell’s disease with thoracolumbar kyphosis who underwent surgical treatment in our department were recruited into this study. All patients were divided into two groups according to whether there were neurological deficits. And the imaging parameters and clinical evaluation indexes of patients before operation, after operation and at the last follow-up were collected and analysed.

Group A included 14 cases of kyphosis with neurological deficits,including 12 females and 2 males, with an average age of 66.8 ± 7.5 years. The mean T value of bone mineral density (BMD) was - 3.3 ± 1.4. As for the neurological function, according to the American Spinal Injury Association (ASIA) impairment scale, 2 cases were classified as B, 5 cases as C and 7 cases as D.

Group B included 16 cases of kyphosis with no neurological deficits, including 13 females and 3 males, with an average age of 64.3 ± 7.7 years; the mean T value of BMD was − 3.6 ± 0.4.

### Inclusion criteria

#### Group A:

1. Meet the diagnostic criteria for Kümmell’s disease, T value of BMD < − 2.5.
2. After conservative treatment for more than 3 months,refractory low back pain still exists.
3. Kyphosis appeared gradually,and it continued to progress. Neurological deficits appeared gradually and aggravated slowly with the progression of kyphosis.
4. Single segment kyphosis with neurological deficits was graded as B-D, according to ASIA impairment scale.

**Group B**: In line with the 1–2 criteria above, thoracolumbar kyphosis is associated with sagittal global/local parameter abnormalities or sagittal imbalance without neurological deficits.

### Exclusion criteria

1. Kümmell’s disease without kyphosis.
2. Severe cardiovascular and cerebrovascular diseases;Diabetes mellitus and other contraindications.
3. Multiple segmental osteoporotic fractures.
4. Kümmell’s disease with old spinal fractures of other segment(s).
5. Patients with lumbar disc herniation,ankylosing spondylitis, spinal tuberculosis, lumbar spondylolisthesis and spinal tumors.
6. Patients who had undergone spinal surgery or vertebroplasty before.

### Imaging evaluation parameters

SVA, Cobb angle, TK,TLK,LL,PI,PT,SS.

### Clinical evaluation indexes

The ODI, NRS, and ASIA grades and complications were all recorded, including infection, deep vein thrombosis (DVT) of lower limbs, cerebrospinal fluid (CSF) leakage, subsidence of internal implants, broken screws and rods, pseudoarthrosis etc. Since it was inconvenient to perform ODI and NRS assessment immediately after surgery, only preoperative assessment and final assessment were performed. Moreover, the recovery of neurological function was relatively slow, so the neurological function of preoperative and final assessment were performed.

### Surgical procedure

**Fig. 1:**
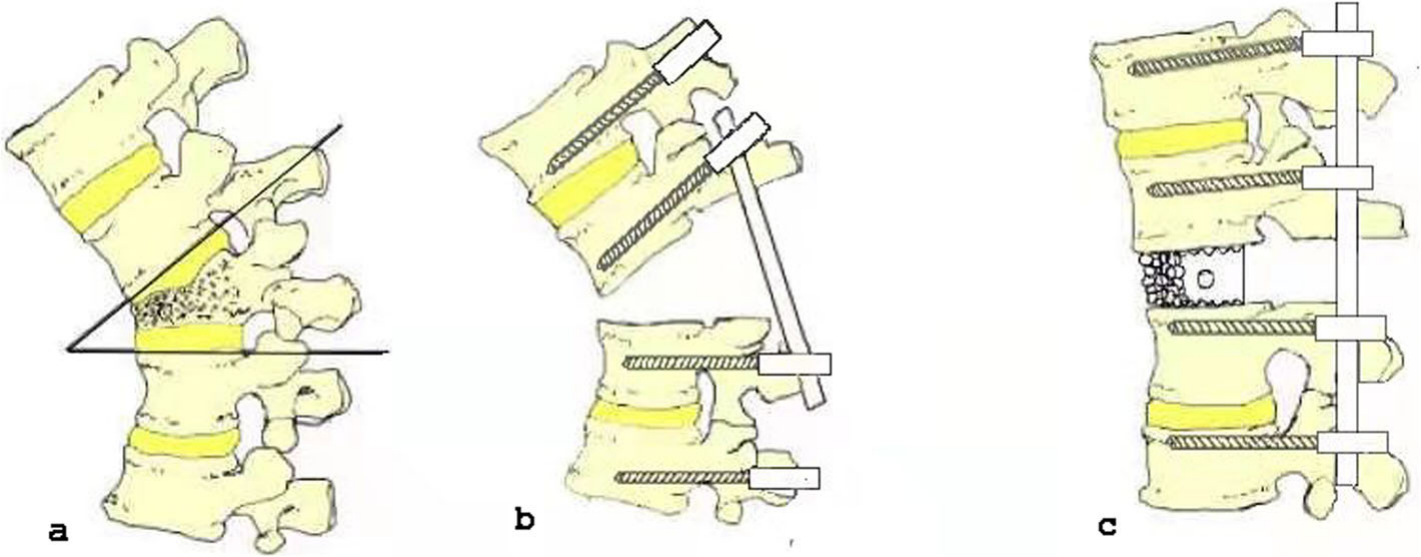
Surgical diagram of group A

Group A: Under general anesthesia and electrophysiological monitoring, the patient was prone on the operating bed. A posterior median incision was performed centering on the injured vertebra. Firstly,pedicle screws were placed in the two upper and two lower vertebrae with the injured vertebra as the center and each screw was strengthened with bone cement. If the injured vertebra was a thoracic vertebra, the proximal ends of bilateral ribs were removed for about 5 cm then the spinous process and the lower 2/3 of the lamina of the upper vertebrae of the injured vertebra were excised. Next the spinous process and lamina of the injured vertebra were excised to expose the spinal canal, protecting the dura and nerve roots. And then the injured vertebra and its upper and lower intervertebral discs were excised. After that, a C-shaped cage made of polyetheretherketone of appropriate size was selected, and autologous bone grains were inserted and placed between the upper and lower end plates of adjacent vertebral bodies. Afterwards the two pre-bent connecting rods were installed, and the cantilever beam technology was used for orthopedic and pressurized locking. During the process of VCR, a temporary rod was used to maintain the spinal stability. Finally, after the drainage tube was placed in the incision, and the incision was closed with layer by layer sutures (Fig. 1).

**Fig. 2:**
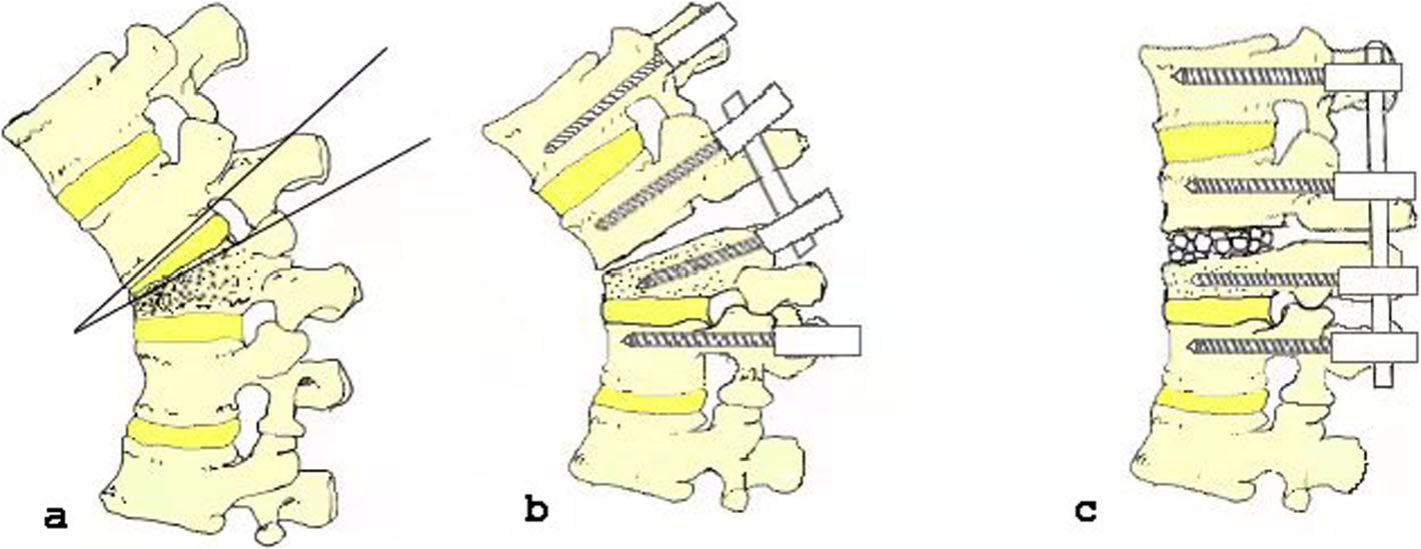
Surgical diagram of group B

Group B: After taking the same procedures of general anesthesia,electrophysiological monitoring as in group A, patients in group B were also exposed with a posterior midline incision. After that,pedicle screws were placed in the two upper and two lower vertebrae on the both sides of the intervertebral space which closed to the collapsed endplate of the injured vertebrae, and the screws were cemented too. Next, the bilateral inferior articular processes of the upper vertebra of the injured vertebra and the bilateral superior articular processes of the injured vertebra were resected. The lower 2/3 lamina of the upper vertebra and the upper 1/3 lamina of the injured vertebra were resected, and the ligamentum flavum was also resected. To protect the nerve roots of both sides, discectomy was performed from the lateral of the dura, and the intervertebral space was released thoroughly. Soon after the upper and lower endplates of the intervertebral space were removed (bone-disc-bone osteotomy), autologous bone granulation grafting in the intervertebral space was performed, and finally the kyphosis was corrected. After the drainage tube was placed in the incision, the incision was closed with layer by layer sutures too (Fig. 2).

Post operation careful attention was paid to the prevention of infection and complications such as deep venous thrombosis of lower extremities.

The drainage tube was removed 3–5 days after the operation, and the patient got out of bed as soon as possible under the protection of braces. Support protection of the brace spanned 3 months. Patients in both groups were treated for osteoporosis after surgical treatment according to the current consensus procedures.

### Statistical methods

Statistical software SPSS21.0 was used for statistical analysis of the above statistical data. The comparison of general data and postoperative and final imaging parameters between the two groups was performed by independent sample T test. One-way ANOVA was used to compare preoperative and postoperative imaging parameters and the last imaging parameters. Paired T test was used to compare ODI and NRS results in groups. Pearson correlation was used to analyze the correlation between the improvement of NRS and the change of sagittal parameters. Setting *P* < 0.05 was statistically significant.

## Results

Group A: The average operative time was 336 ± 60 min, the average operative blood loss was 1125 ± 769 ml, and the average follow-up time was 34.2 months (range 24--50 months). There were 2 cases of CSF leakage, 2 cases of incision infection,1 case of implant subsidence, 1 case of pseudoarthrosis,and 1 case of Proximal Junctional Kyphosis (PJK). In the last follow-up,there was 1 case of Grade C, 3 cases of Grade D and 10 cases of Grade E with Neurological function of ASIA impairment scale, with an average increase of 1.8 levels compared with preoperative level (Table 1).

**Table 1:** Comparison of general data before and after operation between group A and B

Parameters	Group A	Group B	*P* value
Age (years)	66.8 ± 7.5	64.3 ± 7.7	0.440
Male	2	3	
Female	12	13	0.743
T value of BMD	−3.3 ± 1.4	−3.6 ± 0.4	0.628
Operation time (minutes)	336 ± 60	270 ± 48	0.010
Blood loss (ml)	1125 ± 769.3	441.7 ± 159.3	0.007
Preoperative SVA (mm)	75.0 ± 39.2	62.5 ± 26.6	0.386
PreopCobb angle(°)	28.2 ± 6.2	26.5 ± 7.3	0.560
Preop ODI	59% ± 9	44% ± 8	0.000
Preop NRS	4.7 ± 0.9	4.0 ± 1.1	0.225
CSF leakage	2	1	
Incision infection	2	2	
Implant subsidence	1	0	
Pseudoarthrosis	1	1	
PJK	1	0	
DVT	0	1	

Group B: the average operation time was 270 ± 48 min, the average blood loss was 442 ± 159 ml, and the average follow-up time was 38.7 months (range 36-53 months), there was 1 case of CSF leakage, 2 cases of incision infection, 1 case of DVT of lower extremity, and 1 case of pseudoarthrosis. No neurological deficits appeared in group B after operation and at the last follow-up (Table 1).

### Imaging evaluation

In this study, the normal range of SVA was selected as ±40 mm.

**Fig. 3:**
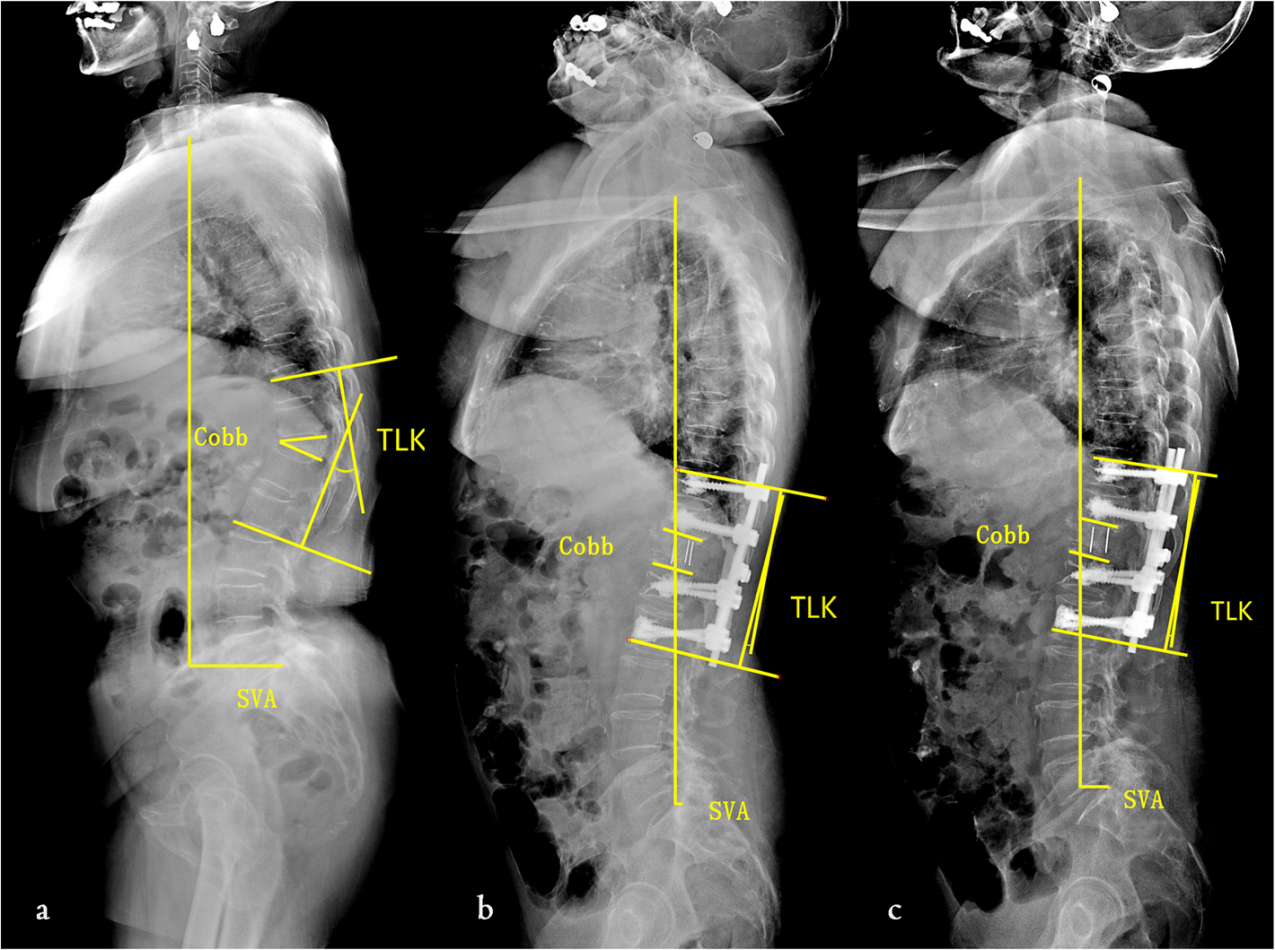
The spinal sagittal parameters of a patient in group A

In Group A:

SVA, Cobb Angle, TLK, PT, ODI and NRS were significantly different among the preoperative, postoperative and last follow-up (*P* < 0.05) (Table 2) (Fig. 3). There was no statistically significant difference in TK, LL, and SS (*P* > 0.05) (Table 2).

**Table 2:** Comparison of data before and after operation and the last follow-up in group A

Parameter	Before operation	After operation	Final follow-up	*p* value
SVA (mm)	75.0 ± 39.2	25.1 ± 17.1	26.7 ± 16.2	0.000
Cobb angle(°)	28.2 ± 6.2	6.4 ± 5.0	7.3 ± 5.3	0.000
TK (°)	34.3 ± 11.7	37.2 ± 10.3	39.0 ± 21.1	0.783
TLK (°)	39.0 ± 21.1	5.7 ± 3.3	7.1 ± 3.1	0.000
LL (°)	45.0 ± 14.3	34.4 ± 7.5	38.4 ± 5.0	0.066
PT (°)	29.0 ± 6.8	20.6 ± 7.7	22.6 ± 8.2	0.048
SS (°)	28.2 ± 9.9	35.6 ± 9.9	36.5 ± 9.2	0.129
ODI	59% ± 9		23% ± 5	0.000
NRS	4.7 ± 0.9		0.9 ± 0.3	0.000

**Fig. 4:**
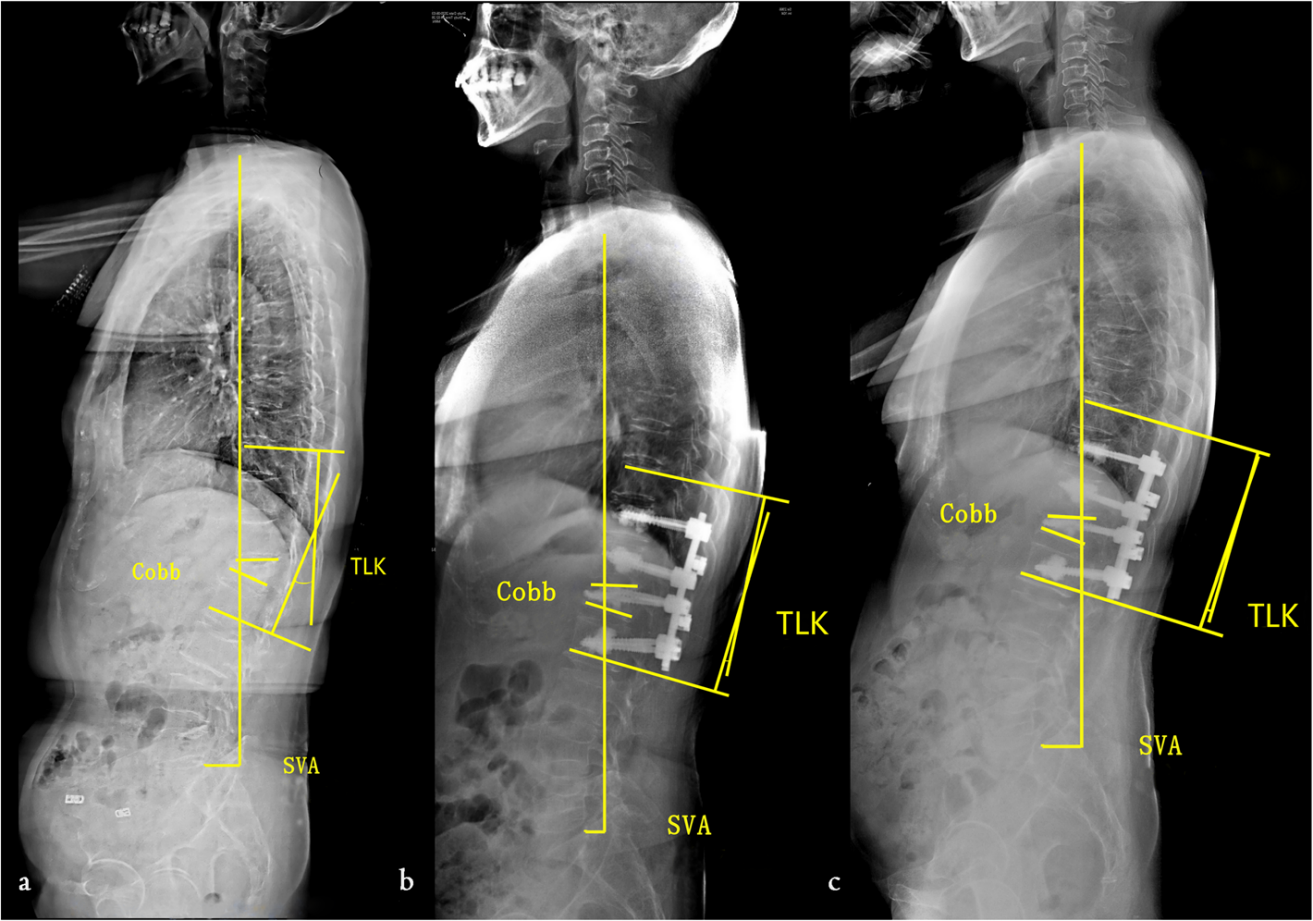
The spinal sagittal parameters of a patient in group B

In Group B:

SVA, Cobb Angle, TLK, ODI and NRS were significantly different among the preoperative, postoperative and final follow-up (*P* < 0.05) (Table 3) (Fig. 4). There was no significant difference in TK, LL, PT, and SS (*P* > 0.05) (Table 3).

**Table 3:** Comparison of data before and after operation and the last follow-up in group B

Parameter	Before operation	After operation	Lastfollow-up	*P* value
SVA (mm)	62.5 ± 26.6	27.2 ± 12.7	30.7 ± 11.6	0.000
Cobb angle(°)	26.5 ± 7.3	15.2 ± 4.7	15.7 ± 4.6	0.000
TK (°)	39.5 ± 15.1	32.1 ± 14.5	36.1 ± 13.7	0.462
TLK (°)	33.0 ± 15.9	7.3 ± 1.3	9.7 ± 1.3	0.000
LL (°)	48.4 ± 14.9	39.3 ± 9.0	42.0 ± 9.3	0.149
PT (°)	17.7 ± 8.4	14.6 ± 5.5	15.1 ± 4.4	0.439
SS (°)	24.9 ± 9.5	29.7 ± 8.9	26.5 ± 10.5	0.470
ODI	44% ± 8		19% ± 5	0.000
NRS	4.0 ± 1.1		0.8 ± 0.6	0.000

Comparison of postoperative imaging results between group A and B: Cobb angle, and PT difference were statistically significant (*P* < 0.05) (Table 4). There were no statistically significant differences in SVA, TK, TLK, LL, and SS (*P* > 0.05) (Table 4).

**Table 4:** Comparison of postoperative imaging results between group A and B

Parameter	Group A	Group B	*P* Value
SVA (mm)	25.1 ± 17.1	27.2 ± 12.7	0.746
Cobb angle(°)	6.4 ± 5.0	15.2 ± 4.7	0.000
TK (°)	34.3 ± 11.7	32.1 ± 14.5	0.710
TLK (°)	5.7 ± 3.3	7.3 ± 1.3	0.140
LL (°)	34.4 ± 7.5	39.3 ± 9.1	0.190
PT (°)	20.6 ± 7.8	14.6 ± 5.5	0.047
SS (°)	35.6 ± 9.9	29.7 ± 8.9	0.156

Comparison of imaging and clinical results at the last follow-up between group A and B: Cobb angle, TLK, PT, SS showed statistically significant differences (*P* < 0.05) (Table 5). There was no significant difference in SVA, TK, LL, ODI, and NRS (*P* > 0.05) (Table 5).

**Table 5:** Comparison of imaging and clinical results at the last follow-up between group A and B

Parameter	Group A	Group B	*P* Value
SVA (mm)	26.7 ± 16.2	30.7 ± 11.6	0.509
Cobb angle(°)	7.3 ± 5.3	15.7 ± 4.6	0.001
TK (°)	37.2 ± 10.4	36.1 ± 13.7	0.833
TLK (°)	7.1 ± 3.2	9.7 ± 1.3	0.014
LL (°)	38.4 ± 5.0	42.0 ± 9.3	0.284
PT (°)	22.6 ± 8.2	15.1 ± 4.4	0.013
SS (°)	36.5 ± 9.2	26.5 ± 10.5	0.028
ODI	23% ± 5	19% ± 5	0.073
NRS	0.9 ± 0.3	0.8 ± 0.6	0.700

The result of correlation analysis showed that the improvement of the NRS of both groups was related to the improvement of the cobb angle (*P* < 0.05). In group A, the improvement of NRS was also positively correlated with the change of PT but negatively correlated with the change of SS (*P* < 0.05) (Tables 6, 7).

**Table 6:** Correlation analysis between clinical outcomes and sagittal parameters of group A

	ΔSVA	ΔCobb angle	ΔTK	ΔTLK	ΔLL	ΔPI	ΔPT	ΔSS
ΔNRS	−0.363	0.773*	0.374	0.251	0.039	−0.618	0.820*	−0.736*
	(*p* = 0.302)	(*p* = 0.009)	(*p* = 0.286)	(*p* = 0.485)	(*p* = 0.914)	(*p* = 0.057)	(*p* = 0.004)	(*p* = 0.015)
ΔODI	0.173	− 0.143	0.518	− 0.304	− 0.07	−0.057	0.429	−0.033
	(*p* = 0.632)	(*p* = 0.694)	(*p* = 0.125)	(*p* = 0.393)	(*p* = 0.838)	(*p* = 0.875)	(*p* = 0.217)	(*p* = 0.927)

*:*p* < 0.05, *ΔNRS* improvement of NRS, *ΔODI* improvement of ODI, *ΔSVA* change of SVA, *ΔCobb angle* change of Cobb angle, *ΔTK* change of TK, *ΔTLK* change of TLK, *ΔLL* change of LL, *ΔPI* change of PI, *ΔPT* change of PT, *ΔSS* change of SS

**Table 7:** Correlation analysis between clinical outcomes and sagittal parameters of group B

	ΔSVA	ΔCobb angle	ΔTK	ΔTLK	ΔLL	ΔPI	ΔPT	ΔSS
ΔNRS	0.363	0.659*	0.269	0.345	0.309	− 0.205	− 0.103	− 0.026
	(*P* = 0.246)	(P = 0.020)	(*P* = 0.398)	(*P* = 0.272)	(*P* = 0.329)	(*P* = 0.522)	(*p* = 0.749)	(*P* = 0.935)
ΔODI	0.500	− 0.568	−0.189	− 0.498	0.291	− 0.458	−0.241	0.151
	(*p* = 0.098)	(*p* = 0.054)	(*p* = 0.555)	(*p* = 0.099)	(p^−0^.359)	(*p* = 0.134)	(*p* = 0.451)	(*p* = 0.639)

*:*p* < 0.05, *ΔNRS* improvement of NRS, *ΔODI* improvement of ODI, *ΔSVA* change of SVA, *ΔCobb angle* change of Cobb angle, *ΔTK* change of TK, *ΔTLK* change of TLK, *ΔLL* change of LL, *ΔPI* change of PI, *ΔPT* change of PT, *ΔSS* change of SS

## Discussion

The authors believe that the balance of the thoracolumbar segment is of great clinical significance for the global balance of the spine, and the restoration of sagittal balance is a key factor in improving quality of life. Therefore, in the treatment of Kümmell’s disease with thoracolumbar kyphosis, to ensure the stabilization of the spine and to relieve the compression to the spinal cord, additional attention should be paid to the restoration of normal thoracolumbar alignment and the correction of sagittal imbalance, so as to avoid or reduce the later refractory low back pain, and to further improve the patient outcomes. Therefore, during the operation, not only the thoracolumbar kyphosis angle of the two groups was restored to the normal range, but also the global balance of the spine sagittal plane was restored. According to Schwab’s classification standard for posterior spinal osteotomy [32], VCR belongs to grade V osteotomy. In group A, after a standard VCR was performed, and with the help of the posterior pedicle internal fixation system, it was relatively easy to correct thoracolumbar kyphosis, to restore normal thoracolumbar alignment and to correct sagittal imbalance. Postoperative results and the last follow-up showed a satisfactory recovery of sagittal parameters.

Our results suggest that in the surgical treatment for cases of kyphosis with no neurological deficits, it may also be important to pay attention to correcting the kyphosis and to regain a good balance of the sagittal plane. By doing so the abnormal stress on the articular process and intervertebral disc can be reduced and fatigue of the back muscles can also be reduced. So in group B, the focus of intraoperative treatment is the release of the intervertebral space on the side of the collapsing endplate of the injured vertebra. The purpose of loosening the gap is to correct local kyphosis and restore sagittal plane balance. Therefore, the bone-disc-bone osteotomy (BDBO) was used in group B. Although BDBO belongs to Schwab IV osteotomy [32], it is not enough to remove part of the upper and lower endplates of the corresponding space to obtain a bone graft bed. This is because, besides the aim above, the range of BDBO should be as large as possible in order to get a spine which is easier to be corrected rather than focusing only on preparation of the bone graft bed. At the same time,after the pedicle screws were cemented, the increased holding force of the screw can increase the strength of the correction, achieve a satisfactory correction in the sagittal plane, and reduce the internal fixation segment.

The results of this study showed that the improvement of the NRS of the two groups was positively correlated with the improvement of the Cobb angle (local kyphosis) in the sagittal plane. In the case of group A, the improvement of NRS was also positively correlated with the change of PT but negatively correlated with the change of SS. It can be seen from the result that while the postoperative Cobb angle improved in the two groups, the local spine alignment was also restored accordingly. We also think that the improvement of the local alignment restored the stress on articular processes, intervertebral discs and other tissues to normal. At the same time, the improvement of the spinal alignment also reduced the work and strain of the back muscles, thereby relieving postoperative back pain. This result is basically consistent with the previous research [22, 23, 26, 31, 33]. In addition, it is considered that sagittal imbalance is an interactive phenomenon that is accompanied with alteration of LL, SS, PI and PT [34]. Therefore, once the sagittal balance is restored, the corresponding sagittal parameters will also change. We speculate that the surgical method of group A was more powerful than that of group B, which caused corresponding changes of PT and SS after the operation. However, the correlation between NRS and changes in PT, and SS needs to be further explored.

Our study had limitations. Firstly, due to the relatively small sample size and short follow-up time in this study the result may introduce some bias, and secondly, the long-term follow-up results of the above cases need to be further demonstrated.

## Conclusion

For Kümmell’s disease of thoracolumbar kyphosis with and without neurological deficits,to restore the normal spinal alignment and sagittal balance can obtain a satisfactory radiographic and clinical short and medium-term effects.

## Data Availability

The datasets generated and/or analyzed during the current study are not publicly available due to limitations of ethical approval involving the patient data and anonymity but are available from the corresponding author on reasonable request.

## Abbreviations

SVA: Sagittal vertebral axis
TK: Thoracic kyphosis
TLK: Thoracolumbar kyphosis
LL: Lumbar lordosis
PI: Pelvic incidence
PT: Pelvic tilt
SS: Sacral slope
ODI: Oswestry disability index
NRS: Numerical rating scale
OVCF: Osteoporotic vertebral compression fracture
VCR: Vertebral column resection
BMD: Bone mineral density
ASIA: American spinal injury association
DVT: Deep vein thrombosis
CSF: Cerebrospinal fluid
PJK: Proximal junctional kyphosis
